# Dexmedetomidine Versus Clonidine as an Adjuvant to Lidocaine Spinal Anesthesia in an Ovine Experimental Model

**DOI:** 10.3390/ani16020197

**Published:** 2026-01-09

**Authors:** Claudia Piemontese, Caterina Vicenti, Alberto Maria Crovace, Roberta Pizzi, Marzia Stabile, Marta Guadalupi, Luca Lacitignola, Francesco Staffieri

**Affiliations:** 1 Department of Precision and Regenerative Medicine and Jonic Area (DiMePRe-J), University of Bari, Piazza Umberto I, 70121 Bari, Italy; caterina.vicenti@uniba.it (C.V.); r.pizzi1@phd.uniba.it (R.P.); marzia.stabile@uniba.it (M.S.); marta.guadalupi@uniba.it (M.G.); luca.lacitignola@uniba.it (L.L.); francesco.staffieri@uniba.it (F.S.); 2 Department of Veterinary Medicine, University of Sassari, Piazza Università, 07100 Sassari, Italy; acrovace@uniss.it

**Keywords:** spinal anesthesia, dexmedetomidine, clonidine, adjuvants, α-2 agonist, sheep, analgesia, pain, sensory blockade, motor blockade

## Abstract

Sheep are routinely used as orthopedic models due to their similarities to human joints. Spinal anesthesia provides adequate analgesia for these procedures, and its duration can be enhanced with adjuvant drugs. Clonidine is commonly used in human spinal anesthesia, while dexmedetomidine is a newer and more selective α-2 agonist. This study compared the duration and analgesic effect of these two drugs as adjuvants in spinal anesthesia. Thirty-nine sheep undergoing experimental pelvic limb cartilage damage surgery were enrolled. Animals were sedated with diazepam (0.4 mg kg^−1^) and buprenorphine (10 μg kg^−1^) intravenously. Propofol was given as needed (0.5 mg kg^−1^) and oxygen support via face mask was continuous. Animals were positioned with the treated limb in a dependent position for the lumbosacral spinal block. Sheep were divided into three groups (*n* = 13), receiving lidocaine 2% (L group), lidocaine 2% + clonidine 20 μg mL^−1^ (CL group), or lidocaine 2% + dexmedetomidine 1 μg mL^−1^ (LD group) for spinal block (1 mL every 10 kg). Recovery times (minute) from the spinal block were recorded: anal sphincter tone (AS), recovery of sensibility (RoS), first limb movements (FMov), time of standing (ToS), and first rescue analgesia; ataxia (ATA) was also measured after standing. Dexmedetomidine increased the duration of spinal anesthesia, affecting both motor and sensory functions.

## Simple Summary

This study compared the effectiveness of two drugs, clonidine and dexmedetomidine, in enhancing the effects of spinal anesthesia. Spinal anesthesia is a technique used to block pain in the lower part of the body during surgery, and it is applied both in people and in sheep undergoing orthopedic procedures on the hind limbs. To make this technique last longer and work more effectively, additional drugs—called adjuvants—are sometimes added. Clonidine and dexmedetomidine belong to a group of drugs called α-2 agonists, which reduce nervous system activity and can improve pain control. This study evaluated the impact of these two drugs on pain relief and mobility. Our findings showed that both drugs provided good pain control during orthopedic surgery. However, dexmedetomidine resulted in a more prolonged loss of sensitivity and mobility in the postoperative period than clonidine. Understanding these differences can help veterinarians and researchers choose the most suitable adjuvant depending on the type of surgery and the expected recovery time. This knowledge may also contribute to refining spinal anesthesia protocols and improving animal welfare in both clinical and experimental settings.

## 1. Introduction

Effective anesthesia and pain management are essential to ensure animal welfare and safety, as well as to minimize stress, risks, and complications. Each phase of anesthesia is crucial not only for animal well-being but also for the success of the procedure and the reliability of the resulting data. In orthopedic research, sheep are widely accepted as an in vivo model for investigating the biomechanical, biochemical, and histological processes of bone biology [1,2,3]. This species shows a high degree of similarity to humans in terms of bone and joint structure, as well as bone remodeling processes [4,5], making it particularly suitable for studies involving bone healing and orthopedic interventions. However, anesthetic procedures in sheep can be associated with unexpected adverse events, including fermentation and ruminal bloating, regurgitation with the risk of aspiration, and pulmonary edema linked to the systemic use of α-2 adrenergic agonists [6]. To reduce or prevent these complications, the implementation of a multimodal anesthesia protocol is strongly recommended. This approach ensures effective, procedure-specific pain management tailored to the individual animal, while simultaneously minimizing the incidence of anesthesia-related complications. In accordance with current legislation on the protection of animals used for scientific purposes (Directive 2010/63/EU), anesthesia is fundamental not only for ensuring animal welfare but also for adhering to the principles of the 3Rs: Replacement, Reduction, and Refinement. Within this context, spinal anesthesia has emerged as a valuable technique for orthopedic procedures—particularly those involving the hind limbs—due to its ability to provide reliable and targeted analgesia [7,8]. This procedure of regional anesthesia involves the direct injection of a local anesthetic into the subarachnoid space, a technique first introduced in the late 1800s. In small ruminants, the subarachnoid space is readily accessible via the lumbosacral intervertebral space (L6–S1), making spinal anesthesia a practical and effective technique in veterinary medicine [9,10]. Intrathecal administration of anesthetic and analgesic agents primarily acts at the dorsal horn of the spinal cord, modulating nociceptive transmission. Several drug classes can induce analgesia when administered intrathecally, including local anesthetics, opioids, α2-adrenergic agonists, and dissociative agents such as ketamine [11,12]. Among these, lidocaine is one of the most widely used local anesthetics for both epidural and subarachnoid anesthesia in small ruminants. It is routinely employed for procedures involving the lower flank, hind limbs, perineum, rectum, and tail. Its mechanism involves blocking voltage-gated Na^+^, Ca^2+^, and K^+^ channels in dorsal horn sensory neurons, resulting in a reversible blockade of sensory, sympathetic, and motor fibers; it has a quick onset and a mean duration of 60–120 min [13,14]. Spinal anesthesia has undergone significant refinement with the development of safer, more selective agents and optimized clinical protocols. The co-administration of adjuvants with local anesthetics has further improved analgesic efficacy and prolonged block duration, representing a notable advancement in perioperative pain management [14]. Intrathecal α2-adrenergic agonists are commonly employed as adjuvants to local anesthetics, as they enhance anesthetic efficacy and permit a reduction in the required dose of the primary agent [15,16]. Clonidine, a partial α2-adrenoreceptor agonist, has been extensively studied for intrathecal use and is associated with a favorable safety and efficacy profile [17]. When combined with local anesthetics, clonidine significantly prolongs the duration of both sensory and motor spinal blockade [15,16,18].

Dexmedetomidine, a highly selective α2-adrenoreceptor agonist used for intravenous sedation and analgesia enhancement, has been found to exhibit an α2/α1 selectivity ratio eight times greater than that of clonidine, suggesting that a potentially enhanced effect could be achieved when it is used intrathecally [19].

The aim of this study is to compare the efficacy of clonidine and dexmedetomidine as adjuvants to lidocaine for spinal anesthesia. Our hypothesis is that dexmedetomidine could be more effective than clonidine in prolonging the sensory and motor blockade of lidocaine. To test our hypothesis, motor, sensory and analgesic effects were monitored in the postoperative period in sheep undergoing orthopedic surgery under spinal anesthesia.

## 2. Materials and Methods

This prospective, randomized, double-blind, experimental study involved 39 sheep undergoing experimental pelvic limb cartilage damage surgery for the application of an osteochondral scaffold and was approved by Ministerial Authorizations n°76/2023-PR and n°263/2023-PR. It was conducted at the Section of Veterinary Clinics and Animal Production of DiMePRe-J at University of Bari “Aldo Moro”, Italy. The sheep were adult females of the Gentile di Puglia breed, weighing 50 kg. They were fasted for 48 h before surgery [20]. After the application of an intravenous (IV) catheter in the auricular vein (DeltaVen 20G, Delta Med S.p.A., Viadana, Italy), the sheep were sedated by administering diazepam (0.4 mg kg^−1^, Ziapam 5 mg mL^−1^, Ecuphar S.R.L., Milano, Italy) and buprenorphine (10 μg Kg^−1^, Bupaq 0.3 mg mL^−1^, Livisto, Modena, Italy) IV. They were positioned on the surgery table on lateral decubitus with the treated limb in a dependent position for the execution of the lumbosacral spinal block. Meanwhile, the head was positioned on a pillow, and a sandbag was placed under the cervical region to slightly elevate it and to allow saliva drainage. A towel was placed over their eyes to keep them calm. The animals were continuously supported with oxygen 100% delivered via face mask (12 L min^−1^) and fluid therapy (Ringer Lactate, Baxter S.p.A., Rome, Italy) at a rate of 5–10 mL kg^−1^ h^−1^. Pre-operative antibiotic and anti-inflammatory therapy were also guaranteed by administering benzilpenicillin + diidrostreptomicin by injection into muscle (5 mL, Repen 200.000 U.I. mL^−1^ + 250 mg mL^−1^, Fatro S.p.A., Ozzano dell’Emilia, Italy) and flunixin (1 mg kg^−1^, Emdofluxin 50 mg mL^−1^, Emdoka, Hoogstraten, Belgium) IV.

During the procedure, the animals were gently restrained, and the depth of anesthesia was assessed using the intraoperative sedation scale, a numerical descriptive scale from 1 (calm and quiet patient requires no additional sedation or restraint) to 3 (patient agitated, moves and requires additional sedation) (Table 1). A dose of 0.5 mg kg^−1^ of propofol (Proposure 10 mg mL^−1^; Boehringer Ingelheim Animal Health Italia S.p.A., Milano, Italy) was given intravenously in cases where the score was 3, in order to ensure an adequate depth of sedation.

For the execution of the spinal block, the sheep were randomly divided into three groups of 13 animals. Randomization was performed using a predefined Excel document (simple random allocation sequence generated with the chit method) based on a 1:1:1 randomization scheme (Microsoft Excel, 16.103.2 software version). Spinal anesthesia solutions were prepared by combining drugs in a 10 mL syringe as follows: 10 mL of lidocaine 2% (Lidor 20 mg mL^−1^, Livisto, Modena, Italy) for the L group; 8.7 mL of lidocaine 2% + 1.3 mL of clonidine (20 μg mL^−1^; Catapresan 150 mcg mL^−1^, Boehringer Ingelheim IT S.p.A., Italy) for the CL group; and 9.98 mL of lidocaine 2% + 0.02 mL of dexmedetomidine (1 μg mL^−1^; Dexdomitor 0.5 mg mL^−1^, Orion corporation, Espoo, Finland) for the LD group. These solutions were prepared by a veterinarian who was solely aware of group allocation and did not participate in intraoperative or postoperative assessments. Prior to the block execution, the lumbosacral area was surgically prepared, and the hind limbs were displaced cranially in order to expose the spine and make the lumbosacral space more evident. After the localization of the iliac crests and the lumbosacral space, the spinal needle (BD Quinke point 20G—0.9 × 90 mm, GIMA S.p.A., Milan, Italy) was advanced midline halfway between the spinous process of the last lumbar vertebra (L6) and the sacrum (S1) until it penetrated the dura-subarachnoid membranes. Then, the stylet was taken out of the spinal needle, and the right placement was confirmed by the free flow of cerebrospinal fluid. At this point, the assigned spinal solution was administered slowly at a dose of 1 mL every 10 kg. The success of the block was assessed by the loss of anal sphincter tone. The animals were kept in lateral decubitus with the treated limb in a dependent position for 10 min. After this time, they were positioned for surgery. During the procedure, heart rate (HR, beats min^−1^), respiratory rate (RR, breaths min^−1^) and non-invasive mean arterial blood pressure (MAP, mmHg) were continuously monitored (Datex-Ohmeda S/5, Vetefarma, Cuneo, Italy) throughout the procedure to assess nociceptive responses, with baseline values determined prior to the start of surgery. An increase of ≥20% in two of these parameters was interpreted as indicative of nociception and prompted the administration of rescue analgesia (fentanyl 2 μg kg^−1^ IV; Fentadon 50 μg mL^−1^, Dechra Veterinary Products Srl, Torino, Italy) [21]. If the monitored variables did not return to within 20% of baseline values within 5 min following the initial bolus, a second dose was administered. The block was deemed unsuccessful if more than three rescue boluses were required. In such cases, the animal was withdrawn from the study and provided with alternative analgesic management consisting of a continuous intravenous fentanyl infusion at a rate of 10 μg kg^−1^ hr^−1^ [22]. In this case, the sheep was intubated and switched to inhalational anesthesia. Temperature (T, °C) was also monitored and a warmer device (BairHugger^TM^, 3M, Pioltello, Italy) was used when necessary to keep it at around 38 °C.

At the end of the procedure, the animals went to the recovery box, and the times (minute) of recovery of anal sphincter tone (AS), the occurrence of the first spontaneous limb movements (FMov), the first attempts to stand (FAtt) and the time of standing (ToS) from the spinal block were recorded. Moreover, the recovery of sensibility (RoS) was evaluated by observing the occurrence of a response to limb pinching with a 25G needle. After standing, the animals were observed every 10 min for one hour and the degree of ataxia (ATA10, ATA20, ATA30, ATA40, ATA50, ATA60) was measured using a numerical descriptive scale (1 = absent; 2 = mild; 3 = severe) (Table 2).

Pain was also assessed using the Sheep Grimace Scale, with a score from 0 to 3 given for each point considering the orbital tightening, the head and ear position, and the presence of flehmen [23]. A Grimace value > 4 was considered a cut-off to require a rescue consisting of IV buprenorphine (10 μg kg^−1^), and the time of its administration (^1st^R) from ToS was recorded.

Sheep received antibiotic and anti-inflammatory therapy during the subsequent days of hospitalization. Following the initial 24 h period after the procedure, buprenorphine was administered in conjunction with the therapeutic regimen, solely as a rescue treatment if the Grimace values indicated this was required.

### Statistics

The sample size was calculated a priori, accepting an alpha risk of 0.05 and a power of 0.9 in a two-tailed test. Twelve subjects were required in each of the three groups to detect a statistically significant difference > 80 min in the time of the first rescue administration from the spinal block. The common standard deviation was assumed to be 60. The calculation was performed using Granmo software, version 8.0 (Institut Municipal d’Investigació Mèdica, Barcelona, Spain). All data were analyzed using MedCalc (12.7.0.0 version) and Jamovi (2.3.28 version) software.

The normal distribution of data was verified using the Shapiro–Wilk test. Intra-operative monitored values, RoS, AS, FMov, FAtt, ToS, and ^1st^R, were calculated as mean ± standard deviation; ATA was calculcated as median and interquartile range (IQR; 25th–75th percentile).

Two-way ANOVA was used to compare the intra-operative monitored values and the time of RoS, AS, FAtt, FMov, ToS, and ^1st^R between groups. The post hoc analysis was performed with the Tukey test. The Kruskal–Wallis test was used to compare boluses of propofol and ATA values between groups. *p* < 0.05.

## 3. Results

The duration of the surgical procedures was 30.6 ± 10.9 min in the L group, 31.1 ± 13.1 min in the LD group, and 30.5 ± 11 min in the CL group. The median number of propofol boluses administered was 3 (IQR: 2–4) in the L group, 3 (2–4) in the LD group, and 3 (2–4) in the CL group. The spinal block technique was easy to perform, requiring a single attempt in all sheep, and none of the animals required additional intraoperative analgesia, confirming the success of the block. The recovery from the block was uneventful, all animals completed the study without complications, and none were excluded. Intraoperative measurements of HR, MAP, RR, T, and peripheral oxygen saturation (SpO_2_) are summarized in Table 3. No statistically significant differences were observed among the three groups for any of these parameters at corresponding time points during the procedure.

**Figure 1:**
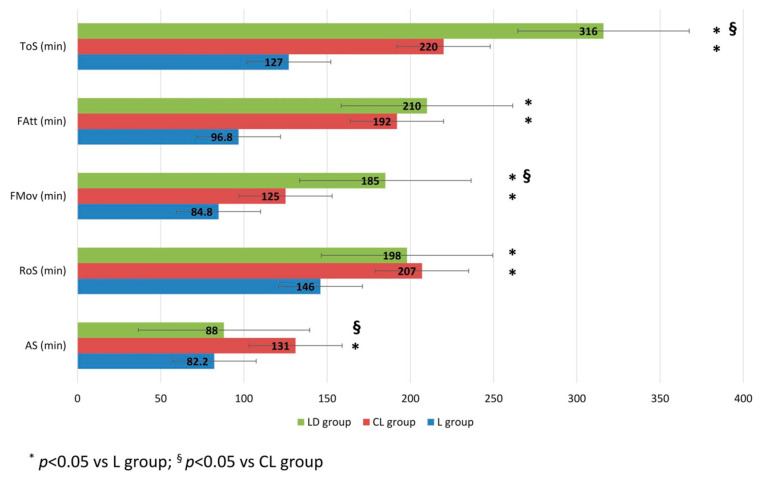
Time of recovery from the spinal block. The times (minute) of recovery of anal sphincter tone (AS), the recovery of sensibility (RoS), the occurrence of the first spontaneous limb movements (FMov), the first attempts to stand (FAtt), and the time of standing (ToS) recorded from the spinal block. (L group: spinal lidocaine 2%, CL group: spinal lidocaine 2% + clonidine 20 μg mL^−1^, LD group: spinal lidocaine 2% + dexmedetomidine 1 μg mL^−1^). * *p* < 0.05 vs. L group; ^§^ *p* < 0.05 vs. CL group.

The recovery time of AS from the spinal block was 82.2 ± 15.1 min in the L group, 131 ± 28 min in the CL group, and 88 ± 51.5 min in the LD group. A statistically significant difference was observed between the CL group and both the L group (*p* < 0.001) and the LD group (*p* = 0.015). The times of FMov were 84.8 ± 29 min in the L group, 125 ± 14.6 min in the CL group, and 185 ± 63 min in the LD group. Statistically significant differences were found among groups (*p* < 0.001 for the L group vs. the CL group and LD group; *p* = 0.03 for the CL group vs. the LD group). The times of FAtt were 96.8 ± 27.9 min in the L group, 192 ± 44.2 min in the CL group, and 210 ± 60.1 min in the LD group, with statistically significant differences between the L group and both the CL group and the LD group (*p* < 0.001). The ToS occurred at 127 ± 25.2 min in the L group, 220 ± 35.7 min in the CL group, and 316 ± 80.3 min in the LD group. A statistically significant difference was found among the three groups (*p* < 0.001). The RoS from the spinal block was 146 ± 21.8 min in the L group, 207 ± 27.1 min in the CL group, and 198 ± 60.4 min in the LD group, with a statistically significant difference for the L group compared to the other two groups (*p* < 0.001 for the L group vs. the CL group and *p* < 0.008 for the L group vs. the LD group). These data are illustrated in Figure 1.

**Figure 2:**
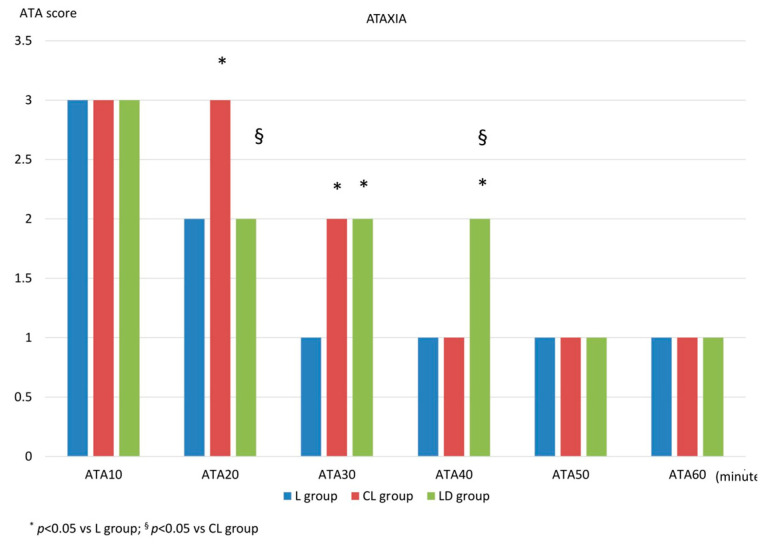
Ataxia score after standing. After standing, the degree of ataxia was measured using a numerical descriptive scale, 1 = absent; 2 = mild; 3 = severe, every 10 min for one hour (ATA10, ATA20, ATA30, ATA40, ATA50, ATA60). L group: spinal lidocaine 2%, CL group: spinal lidocaine 2% + clonidine 20 μg mL^−1^, LD group: spinal lidocaine 2% + dexmedetomidine 1 μg mL^−1^. * *p* < 0.05 vs. L group; ^§^ *p* < 0.05 vs. CL group.

After standing, at ATA10, all three groups exhibited severe ataxia, with a score of 3, and no statistically significant differences were observed among the groups. At ATA20, the score was 2 (2-1) for the L group, 3 (3-2) for the CL group, and 2 (2-2) for the LD group, with a statistically significant difference for the CL group compared to both the L group (*p* < 0.001) and the LD group (*p* = 0.02). At ATA30, the score was 1 (2-1) for the L group, 2 (3-2) for the CL group, and 2 (2-2) for the LD group. At this time point, a statistically significant difference was found between the L group and the other two groups (*p* < 0.001 vs. the CL group; *p* = 0.004 vs. the LD group). At ATA40, the score was 1 (1-1) for the L group, 1 (2-1) for the CL group, and 2 (2-2) for the LD group, with a statistically significant difference observed for the LD group compared to the other two groups (*p* < 0.001). At ATA50 and ATA60, all groups reached a score of 1 (1-1), with no statistically significant differences among them. These data are illustrated in Figure 2.

**Figure 3:**
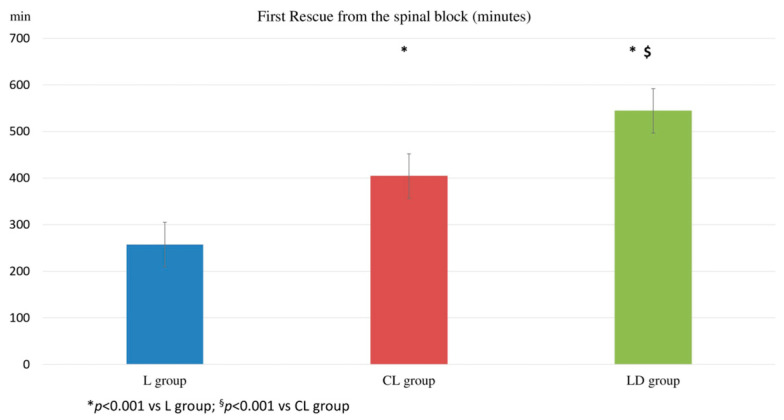
First rescue administration from the spinal block. The time (minute) of the first rescue analgesia administration from the spinal block. (L group: spinal lidocaine 2%, CL group: spinal lidocaine 2% + clonidine 20 μg mL^−1^, LD group: spinal lidocaine 2% + dexmedetomidine 1 μg mL^−1^.) * *p* < 0.001 vs. L group; ^§^ *p* < 0.001 vs. CL group.

The first analgesic administration (^1st^R) after the spinal block was 257.2 ± 65.7 min in the L group, 404.2 ± 47.7 in the CL group, and 544.2 ± 84.2 min in the LD group (*p* < 001). These data are illustrated in Figure 3.

## 4. Discussion

This study demonstrated that spinal anesthesia, whether performed with lidocaine alone or in combination with the adjuvants clonidine or dexmedetomidine, can be considered an effective and reliable analgesic strategy for experimental orthopedic surgeries involving the hind limbs. Across all three treatment groups, intraoperative hemodynamic parameters remained stable, and none of the animals required additional intraoperative analgesia, confirming the success of the spinal block.

In the postoperative phase, animals in the LD group exhibited a longer recovery of motor function and a prolonged ataxic phase compared to those in the L and CL groups. Notably, the LD group also showed a delayed onset of pain following standing and consequently required rescue analgesia at a later time point compared to the other groups.

The most relevant outcome of this study concerns the analgesic effect, which was of longer duration in the groups receiving α2-agonist adjuvants. These findings are consistent with other studies in sheep and other species, showing that the analgesic effect of α2-agonists is related to both a direct action on the spinal cord and an “indirect” synergistic effect with the local anesthetic. Several studies in human medicine have evaluated dexmedetomidine and clonidine as adjuvants in spinal blocks with various local anesthetics, reporting that dexmedetomidine significantly prolongs the duration of motor and sensory blockade and delays the time to first analgesic request [24,25,26]. Multiple mechanisms may underlie the analgesic action of these agents, including direct stimulation of α2-adrenoreceptors in the dorsal horn of the spinal cord, which inhibits substance P and other nociceptive neurotransmitters, modulation of cholinergic pathways, alteration of nociceptive impulse transmission, and both direct local anesthetic-like effects and potentiation of co-administered local anesthetics [27,28,29,30,31]. The longer duration of both sensory and motor blockade observed in the LD group compared to the CL group may be explained by the differing pharmacological profiles of dexmedetomidine and clonidine. Clonidine, a partial α2-adrenoreceptor agonist, provides analgesia primarily through inhibition of C-fiber neurotransmitter release and hyperpolarization of postsynaptic neurons in the dorsal horn [27]. In contrast, dexmedetomidine is a second-generation, highly selective α2A-adrenoceptor agonist, which provides greater analgesic potency at lower doses due to its higher receptor selectivity—approximately eightfold that of clonidine [27,32,33]. This difference was further reflected in the postoperative phase. In addition, studies in other veterinary species have highlighted the synergistic effect of dexmedetomidine with local anesthetics, not only when administered at the site of local anesthetic injection but also systemically via intramuscular or intravenous routes [34,35].

Bradycardia and hypotension have been reported as side effects following intrathecal administration of α2-agonists in humans [36,37]. In the present study, none of the treatment groups exhibited these hemodynamic alterations. Eisenach and Tong (1991) investigated the hemodynamic effects of clonidine administered intrathecally at different spinal levels (cervical, thoracic, and lumbar) in sheep [38]. Their results showed that the most pronounced hypotensive effects occurred following cervical and thoracic administration, whereas lumbar administration caused only a mild decrease in heart rate. Moreover, they observed a dose-dependent relationship, with more significant hemodynamic changes at higher doses of clonidine. Eisenach et al. (1994) reported hypotension following intrathecal administration of 100 μg of dexmedetomidine; in contrast, our study used a dose of 5 μg per sheep [39]. These differences in dosage, along with the lumbar site of injection, may explain the hemodynamic stability observed in our study when using clonidine and dexmedetomidine as adjuvants.

Regarding motor blockade, the time of standing was prolonged in groups receiving α2-agonists, which was associated with more severe ataxia. Several studies on intrathecal administration of α2-agonists in sheep and goats have reported prolonged sternal recumbency and persistent ataxia in animals receiving α2-agonists combined with local anesthetics [7,40]. This phenomenon has been attributed to secondary central effects on the locus coeruleus, likely due to systemic absorption through vascular and lymphatic pathways. These effects include sedation and muscle relaxation, in addition to the primary local anesthetic action at the spinal level [40]. The findings of the present study are consistent with these observations.

It is important to note that prolonged recovery, characterized by extended sternal recumbency and persistent ataxia, may pose significant risks. Potential complications include myopathies, neuropathies, excessive ruminal fermentation, or trauma during the ataxic phase, especially if the animals are not adequately restrained [41]. These aspects may represent a limitation not only in an experimental setting but also in clinical contexts, particularly when procedures are of short duration and early ambulation is desirable—for example, during minor interventions—or when prolonged postoperative monitoring or appropriate containment facilities are not available. In the present study, for reasons related to the surgical protocol, the animals were monitored for three months following the intervention, and once spinal blockade and ataxia had resolved, no adverse effects attributable to the intrathecal administration of adjuvants were observed throughout the follow-up period.

Furthermore, based on the findings of this study, the selection of clonidine or dexmedetomidine as intrathecal adjuvants could be more effectively tailored to the nature and the expected duration of the surgical procedure. Dexmedetomidine could be the drug of choice for extended surgeries, but its administration demands close postoperative monitoring to manage the risk related to prolonged ataxia. In contrast, when minimizing motor impairment and facilitating recovery are priorities, clonidine could represent a more appropriate option, while still providing satisfactory postoperative analgesia.

## 5. Conclusions

Spinal anesthesia proved to be an effective technique for experimental orthopedic procedures in sheep, with lidocaine alone being effective for short procedures, lasting around 30 min. Additionally, clonidine and dexmedetomidine were found to be effective intrathecal adjuvants in prolonging motor and sensory blockade, as well as enhancing postoperative analgesia. However, their different pharmacodynamic profiles may influence clinical decision-making. Dexmedetomidine could be more suitable for longer procedures due to its prolonged effects, though it may require closer postoperative monitoring to manage extended ataxia and recumbency. In contrast, clonidine could offer a safer alternative when faster recovery and reduced motor impairment are desired, without compromising postoperative analgesia. These findings highlight the importance of tailoring adjuvant selection to the specific surgical context and recovery needs. Moreover, the use of these α-2 agonists as adjuvants may contribute to the refinement of spinal anesthesia protocols, enhancing the balance between anesthetic efficacy, animal welfare, and postoperative management.

## Figures and Tables

**Table 1:** Intraoperative sedation score. Score used to evaluate the degree of sedation of the animals during the procedure. A bolus of propofol at a dose of 0.5 mg kg^−1^ was administered when the score was 3.

Score	Description
**1**	Calm and quiet patient requires no additional sedation or restraint
**2**	Patient requiring minimum physical restraint
**3**	Patient agitated, moves and requires additional sedation

**Table 2:** Ataxia Score (ATA). The numerical descriptive scale used to assess the degree of ataxia after standing.

Score	Description
**1**	**Absent**, when the patient could easily maintain the quadrupedal position and load weight on all four limbs
**2**	**Mild**, if the patient maintained the station, presented weakness in the hind limbs, could walk but staggering noticeably
**3**	**Severe**, if the patient had severe weakness in their hind limbs, could hold the standing position for a few minutes before falling backwards

**Table 3:** Physiological values recorded during anesthesia. Mean ± standard deviation of heart rate (HR, bpm), respiratory rate (RR, brpm), mean arterial pressure (MAP, mmHg), peripheral arterial blood oxygen saturation (SpO_2_, %), and temperature (T, °C) recorded from premedication (PREMED) to the end of the procedure every 10 min for each group (L group: spinal lidocaine 2%, CL group: spinal lidocaine 2% + clonidine 20 μg mL^−1^, LD group: spinal lidocaine 2% + dexmedetomidine 1 μg mL^−1^).

	GROUP	PREMED	T10 (SPINAL)	T20	T30	T40
**HR**	L group	127.7 ± 37.9	125 ± 29.6	114.4 ± 25.2	113 ± 28.3	115.5 ± 24.8
(bpm)	CL group	125.3 ± 20.9	123.2 ± 21.9	120.5 ± 15.5	112.4 ± 18.4	104.3 ± 14.9
	LD group	128.5 ± 24.8	126.5 ± 19.7	111.7 ± 25.2	115 ± 20.3	111.1 ± 9.4
**MAP**	L group	92.5 ± 34.9	91.2 ± 21.8	97.2 ± 20.7	84.6 ± 16.6	91 ± 15
(mmHg)	CL group	98.7 ± 7.2	100.6 ± 15.2	92.1 ± 6.2	96.3 ± 11.3	92.7 ± 7.3
	LD group	77 ± 9.8	79.5 ± 13.9	87.2 ± 10.2	86.2 ± 8.9	78.2 ± 4.8
**SpO_2_**	L group	95.6 ± 2.8	95.1 ± 2.5	95 ± 3.6	94.7 ± 2.9	95.5 ± 3.6
(%)	CL group	92.6 ± 2.5	92.3 ± 2.3	92.9 ± 2.1	91.5 ± 4.1	93.8 ± 3.2
	LD group	95.7 ± 4.6	95.6 ± 4	95.1 ± 3.9	94.3 ± 4.6	94.4 ± 6.1
**RR**	L group	22.6 ± 6.4	21.3 ± 7.3	20.5 ± 6.7	20.3 ± 6.5	21.7 ± 4.9
(brpm)	CL group	26.3 ± 1.4	22.6 ± 4.2	22.4 ± 3.3	23.8 ± 2.9	23.4 ± 3.3
	LD group	24.9 ± 6.3	21.8 ± 5.8	21.1 ± 4.3	20.9 ± 5.7	21.8 ± 7.9
**T**	L group	38.5 ± 0.4	38.3 ± 0.4	38.2 ± 0.25	38.2 ± 0.3	37.9 ± 0.1
(° C)	CL group	38.9 ± 0.2	38.5 ± 0.5	38.5 ± 0.4	38.3 ± 0.5	38.1 ± 0.5
	LD group	37.6 ± 0.6	38	38	37.7 ± 0.5	37.6 ± 0.6

## Data Availability

The original contributions presented in this study are included in the article. Further inquiries can be directed to the corresponding author.

## Abbreviations

The following abbreviations are used in this manuscript:

L group	Lidocaine group
CL group	Lidocaine + clonidine group
LD group	Lidocaine + dexmedetomidine group
AS	Return of anal sphincter tone
RoS	Recovery of sensibility
FMov	First limb movements
ToS	Time of standing
ATA	Ataxia
IV	Intravenous
HR	Heart rate
RR	Respiratory rate
MAP	Mean arterial blood pressure
T	Temperature
Fatt	First attempt to stand
^1st^R	First rescue analgesia administration
ANOVA	Analysis of variance
IQR	Interquartile range
SpO_2_	Peripheral oxygen saturation
PREMED	Premedication
